# THE ASYMMETRIC EFFECTS OF THE INCREASE AND DECEASE IN INTERGENERATIONAL TRANSFERS ON DEPRESSIVE SYMPTOMS

**DOI:** 10.1093/geroni/igae098.1488

**Published:** 2024-12-31

**Authors:** Sujeong Park, Jinho Kim

**Affiliations:** Korea University, Seoul, Republic of Korea; Korea University, Seoul, Republic of Korea

## Abstract

Despite the existing body of research on the dynamics of intergenerational transfers, there remains a gap in understanding whether decreases and increases in such transfers have differential effects on depressive symptoms among older Korean adults. This study aims to address this gap by examining two key

**objectives:**

(a) exploring the asymmetric effect of intergenerational transfers from children to parents on parental depressive symptoms and (b) investigating whether this asymmetric effect differs by gender. Using seven waves of the Korean Longitudinal Study of Ageing (KLoSA), a novel asymmetric fixed effects model was employed to separately estimate the association for decreases and increases in intergenerational transfers. Our analysis, based on standard fixed effects estimates, uncovered a negative association between intergenerational transfers and depressive symptoms among older adults in Korea. This association was found to be significant solely for mothers, with no significant effect observed for fathers. Moreover, our findings suggest that the asymmetric associations between intergenerational transfers and depressive symptoms are more pronounced for women than for men. Specifically, the asymmetric fixed-effects models revealed that a decrease in intergenerational transfers was linked to increased depressive symptoms among women, whereas an increase in transfers did not show a significant association with depressive symptoms. These results indicate a stronger emotional bond between mothers and their adult children within the Korean context. Given the asymmetric effect of intergenerational transfers, this study contributes to our understanding of the gendered dynamics of intergenerational relationships and their implications for mental well-being among older adults.

